# Ligand Recognition by the Macrophage Galactose-Type C-Type Lectin: Self or Non-Self?—A Way to Trick the Host’s Immune System

**DOI:** 10.3390/ijms242317078

**Published:** 2023-12-03

**Authors:** Justyna Szczykutowicz

**Affiliations:** Department of Biochemistry and Immunochemistry, Division of Chemistry and Immunochemistry, Wroclaw Medical University, Sklodowskiej-Curie 48/50, 50-369 Wroclaw, Poland; justyna.szczykutowicz@umw.edu.pl

**Keywords:** MGL, CLEC10A, tolerance, immune evasion, Tn antigen, LacdiNAc, immune suppression, metastatic cancer cells, fertilization, pregnancy

## Abstract

The cells and numerous macromolecules of living organisms carry an array of simple and complex carbohydrates on their surface, which may be recognized by many types of proteins, including lectins. Human macrophage galactose-type lectin (MGL, also known as hMGL/CLEC10A/CD301) is a C-type lectin receptor expressed on professional antigen-presenting cells (APCs) specific to glycans containing terminal GalNAc residue, such as Tn antigen or LacdiNAc but also sialylated Tn antigens. Macrophage galactose-type lectin (MGL) exhibits immunosuppressive properties, thus facilitating the maintenance of immune homeostasis. Hence, MGL is exploited by tumors and some pathogens to trick the host immune system and induce an immunosuppressive environment to escape immune control. The aims of this article are to discuss the immunological outcomes of human MGL ligand recognition, provide insights into the molecular aspects of these interactions, and review the MGL ligands discovered so far. Lastly, based on the human fetoembryonic defense system (Hu-FEDS) hypothesis, this paper raises the question as to whether MGL-mediated interactions may be relevant in the development of maternal tolerance toward male gametes and the fetus.

## 1. Introduction

Cells, as well as numerous macromolecules in nature, carry an array of simple and complex carbohydrates attached to a protein or lipid core. Interestingly, the high diversity of glycans allows for biological information to be contained in these structures. Such sugars may act as specific markers on the surfaces of cells or macromolecules. The information stored in a carbohydrate structure is deciphered and transferred to the appropriate biological activity via protein–glycan interactions. Sugars may be recognized by many types of proteins, such as enzymes, antibodies, and lectins. In living organisms, there are many different endogenous lectins, including those that support the functions of the immune system through the recognition of foreign antigens. These lectins have evolved to differentiate “self”, “non-self”, “damaged self”, or “altered self” ligands and influence the outcome of the immune response. So far, several types of human immune system lectins have been described, including I-type lectins (Siglecs), galectins, and C-type lectin receptors (CLR) [1,2,3]. The latter bind ligands in a Ca^2+^ dependent manner and are assigned the function of effective antigen receptors on dendritic cells (DCs)—specialized antigen-presenting cells (APCs)—and macrophages [4,5,6]. Among the best-studied DC-expressed receptors of the CLR family are DC-SIGN (dendritic cell-specific intercellular adhesion molecule-3-grabbing non-integrin, also known as CLEC4L/CD209), MR (mannose receptor, also known as CLEC13D/CD206), dectin-1 (also known as CLEC7A/CD369), and MGL (macrophage galactose-type lectin, also known as CLEC10A/CD301), which are all capable of internalizing, processing, and presenting glycosylated antigens in an MHC-restricted manner and mediating the development of an appropriate immune response [7,8]. CLR engagement may activate distinct signaling pathways affecting gene expression and the synthesis of specific molecules and cytokines, resulting in the regulation of APC function and, hence, the type of immune response. Since DC-expressed CLRs are able to positively or negatively instruct DC differentiation, they are important players in immunity modulation [4].

**Figure 1:**
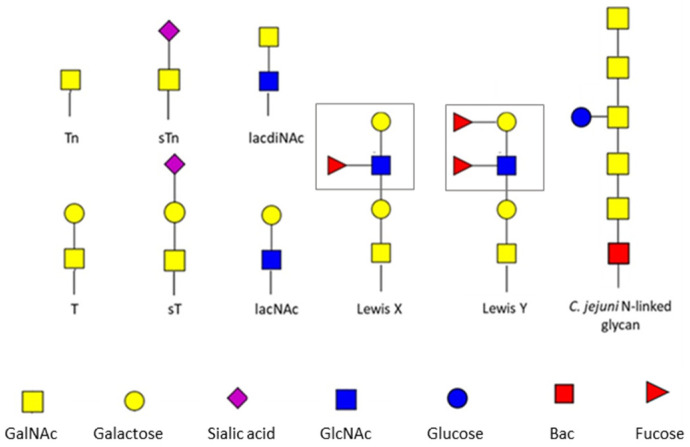
A schematic representation of glycan structures.

In the last two decades, MGL has increasingly attracted the attention of researchers, especially in the context of cancer progression. This receptor was found in the late 1980s on mouse macrophages [9,10]. Further extensive study revealed two related lectins in mice, one mainly expressed by macrophages (MGL1) and the other (MGL2) predominantly expressed by DCs [11,12]. In humans, MGL receptor was found as being homologous to the mouse MGL2 [13,14]. In the human immune system, MGL is the only C-type lectin receptor that recognizes terminal N-acetylgalactosamine (GalNAc) residues, including Tn antigen (αGalNAc-Ser/Thr) and the LacdiNAc (GalNAcβ1-4GlcNAc) epitope but also sialylated Tn antigen [15,16,17]. In mice, MGL2 exhibits the specificity of human MGL with an additional capacity to bind terminal galactose moieties, whereas MGL1 is highly specific to the Lewis X (Galβ1–4(Fucα1–3)GlcNAc) structure (all the glycan structures mentioned are presented in Figure 1). However, this article primarily focuses on the human MGL ortholog [15,17]. In healthy human tissues, the Tn antigen is typically masked by sugars that occur sequentially in the elongated and more diverse O-glycan structures [18,19,20,21,22]. Under physiological conditions, MGL binding to CD45 on effector T cells leads to lymphocyte dampening or even apoptosis [23,24]. Moreover, MGL is overexpressed by APCs with tolerogenic phenotype, which indicates the important role of MGL in the mechanisms of immunosuppression and immune tolerance development. It is thought that the primary function of MGL is to protect the organism from excessive inflammation and autoimmune diseases [8,24,25]. However, the MGL ligand structure may be important for triggering signal transduction and promoting a particular immunological outcome. Depending on the density of glycans and the length and steric structure of the ligand, MGL engagement may also redirect the recognized glycoepitope to the presentation pathway via MHC class I and II, resulting in a CD4+ and CD8+ T cell response [17].

Although C-type lectin receptors can uptake foreign antigens for processing and presentation, some pathogens and metastatic cancer cells exploit interactions with CLRs, including MGL, as survival strategy. They have learned to use CLRs to reduce the antigen presentation capacity of DCs, modulate naïve T cell priming, and induce an immunosuppressive environment, thus escaping immune control [8,26].

Glycan-mediated interactions also seem to be important for survival at early stages of human development. According to the human fetoembryonic defense system (Hu-FEDS) hypothesis, the mechanisms involved in the achievement of immune tolerance during fertilization and pregnancy are similar to those that pathogens and metastatic cancer cells use to evade host immune surveillance and are based on carbohydrate recognition [27,28,29,30,31]. Components of seminal plasma or pregnancy-related glycoproteins have been described as ligands of several lectins of the immune system [32,33,34]. There are no reports as to whether they interact with MGL, although there are some interesting data suggesting that such binding may occur at various stages of fertilization and pregnancy.

The aim of this work is to highlight immunological outcomes of MGL ligand recognition, provide insight into the molecular aspects of these interactions, review the MGL ligands discovered so far, and, lastly, pose a question as to whether MGL interactions are possibly relevant for fertilization and pregnancy.

## 2. Immunological Effect of Ligand Recognition by MGL

### 2.1. MGL as a Guardian of Immune Homeostasis

MGL is most highly expressed on immature DCs (iDCs), monocyte-derived DCs (moDCs), CD1c+ DCs as their specific marker, and alternatively activated macrophages (subtype M2a), and its level may depend on various factors and the local microenvironment [14,35,36,37,38,39]. Zizzari et al. [39] found that the variation in MGL expression depends on seasonal changes with a negative trend that dropped to 33% during the summer and increased to 100% in winter. In turn, van Vliet et al. [25] observed MGL upregulation on tolerogenic DCs in the presence of immunosuppressive dexamethasone and during chronic inflammatory conditions such as rheumatoid arthritis. MGL upregulation on tolerogenic DCs contributes to dampening T cell immunity in an MGL-dependent manner through interacting with a Tn antigen on the CD45 molecules of effector T cells. This binding suppresses the phosphatase activity of CD45 and inhibits lymphocyte protein tyrosine kinase (Lck) activation. In effector T cells, CD45-mediated dephosphorylation of the C-terminal tail of Lck leads to the formation of its active form, which in turn is required for the initiation of T cell receptor (TCR) signaling [40,41]. Therefore, the suppression of CD45 activity by MGL reduces TCR signaling pathways, thus leading to the inhibition of T cell proliferation, reduction in pro-inflammatory cytokine synthesis, and, therefore, the acceleration of T cell apoptosis [17,24,42]. On the other hand, in DCs expressing MGL, binding to the Tn antigen augments the signal transduction pathway involving extracellular signal-regulated kinase 1,2 (ERK 1,2) mitogen-activated protein kinase (MAPK) phosphorylation, which triggers downstream targets—p90RSK and CREB. This action leads to an increase in the secretion of toll-like receptor (TLR)-induced IL-10 and TNF-α. [43,44,45,46]. According to some reports, MGL also activates the nuclear factor-κB (NF-κB) pathway, which seems to be crucial for the production of an increased level of IL-10 [45]. However, findings on MGL-mediated signaling pathway activation are often contradictory [37,43,44,45,46]. IL-10 is an anti-inflammatory cytokine, and interaction with its receptor, IL-10R, on T cells results in the expression of genes, promoting an anti-inflammatory response [23,38,47]. An elevated level of IL-10 has been associated with the differentiation of regulatory T cells (Treg), having an established role in the silencing of an excessive immune response and maintaining homeostasis [48,49]. Also, TNF-α, although classified as a pro-inflammatory cytokine, may exhibit suppressive properties when accompanied with IL-10 [50].

Together, this evidence indicates that MGL plays an important role as a regulator of immune homeostasis and is an agent capable of suppressing immune response. Interestingly, interactions mediated by MGL may have an impact on both the cells bearing MGL ligands and the cells expressing lectin.

### 2.2. MGL Ligands—Players with Different Faces

**Figure 2:**
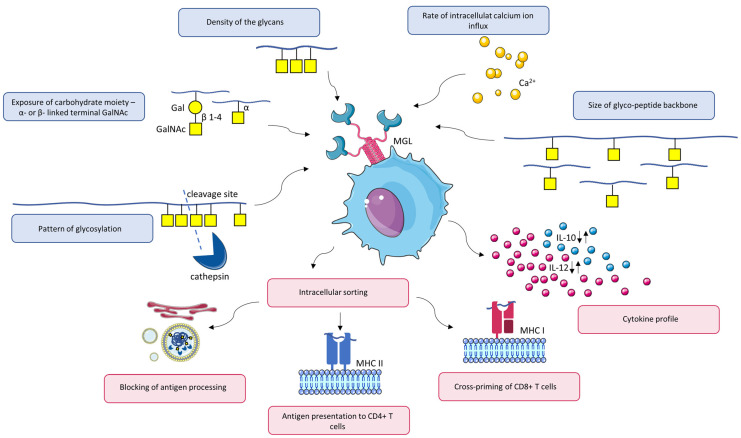
Different contexts of MGL ligands and the possible immunological outcomes of their binding. Images were taken from Servier Medical Art (https://smart.servier.com (accessed on 4 August 2023)) and modified by the authors under the following terms: Creative Commons Attribution 3.0 Unported License.

Even though the primary function of MGL is considered to be immune suppressive rather than pro-inflammatory, a more recent study suggested that the nature of the recognized ligand may be important for the activation of the appropriate molecular pathway and the induction of a particular immune profile. It has been speculated that small changes in the structure can bring about a thoroughly different immune outcome (Figure 2). Among other things, the means of exposure of the GalNAc moiety and glycosidic bond seem to be significant. Diniz et al. [51] reported that, depending on the binding of terminal α- or β-linked GalNAc residues, strong changes are caused in MGL conformation. As signal transduction through CLRs is generally accompanied by their structural alterations, such a result may raise the hypothesis that the distinct anomeric configuration and context of the GalNAc moiety may affect various signaling cascades and, as a consequence, induce different DC phenotypes. These claims were in line with a study by Zaal et al. [52]. They employed two different terminal GalNAc-containing epitopes and observed a distinct effect of MGL binding on the TLR-induced production of immunosuppressive IL-10. Strikingly, GalNAcβ1-4Gal dendrimers were able to augment IL-10 production, whereas MGL engagement with αGalNAc did not induce this effect. Moreover, the analysis of MGL ligation impact on DC biology at the transcriptional level demonstrated that GalNAcβ1-4Gal affects significantly more transcripts than αGalNAc, which had only minimal effects on the transcriptome of DCs; this will be discussed in more detail in the next section of the article.

As a C-type lectin receptor, MGL exhibits endocytic activity and is able to internalize soluble Tn-bearing antigens whose intracellular route and further fate depend on their structure [14,17,37,38]. After endocytosis into DC, the recognized ligand dissociates from the coupled CLR receptor, under the appropriate endosomal environment. Following release, the receptor may be recycled back to the cell surface or degraded, whereas the antigen, depending on its properties, is further trafficked to different intracellular compartments [53]. In general, ingested ligands may be localized within endolysosomal compartments for MHC II presentation or targeted for loading onto MHC I complexes and cross-presentation via the vacuolar or endosome-to-cytosol route [53,54,55,56]. Napoletano et al. [57] found that the binding of tumor-associated mucin 1 (MUC1) peptides carrying a Tn antigen by MGL-expressed on immature monocyte-derived DCs leads to the internalization of recognized glyco-antigens, which may be further delivered into both MHC class I and II compartments. However, this intracellular sorting depends on the size of MUC1 glyco-peptides and its glycosylation pattern. The study showed that MGL-internalized 60 amino acid peptides (corresponding to the 3 tandem repeats of MUC1) are capable of being processed through the MHC class II and I pathways, which can result in glyco-antigen presentation to CD4+ T cells or the cross-priming of CD8+ T cells, respectively. Interestingly, an analysis of the interaction of MGL with the larger soluble glycoprotein of the recombinant MUC1, composed of 16 tandem repeats corresponding to those found in vivo in tumor tissues, showed that the glycoprotein enters the MHC II but does not reach the MHC I compartments. Although cancer-related MUC-1 is only available in full-length form in vivo, MUC-1-specific CD8+ T cells were found in patients with cancer [58,59]. However, this effect may be due to another sugar-independent mechanism of antigen uptake [60].

It should also be considered that intracellular sorting may also depend on the glycosylation of particular serine and threonine residues within the tandem repeats of MUC1. High levels of glycans may inhibit protein degradation through lysosomal cathepsin L by covering specific cleavage sites [54,61]. This results in inability to process and present the antigen. In addition to lysosomal cleavage, the glycosylation pattern also affects the architecture of the antigen that is presented. There is evidence that both CD4+ and CD8+ T cells can recognize peptides devoid of glycans as well as glycan-linked peptides in an MHC-restricted manner [62]. Glycan recognition by primed T-cells occurs if a sugar structure is attached to a suitable site on the peptide. Thus, considering MHC class II, if the glycan structure is located outside the peptide binding core of the MHC molecule, it will not be recognized by T cells [62,63]. However, if the glycan group is located within the MHC binding core, such recognition is possible, provided the glycan is not attached to MHC anchor amino acids; otherwise, the glycopeptide will not be able to bind to the MHC and will thus be non-immunogenic. If the glycan is attached to the central position of the MHC core, far from the MHC anchor, the glycan becomes the dominant part of the epitope, which may be later recognized by specific T cells [64]. Still, not all glycans attached in this way elicit an immune response. It is probable that large and highly complex glycans cannot be accommodated in the central region of TCR [65,66]. In response to glycoproteins containing glycan structures in the central MHC core (e.g., short O-linked GalNAc), T cells specific to the non-glycosylated cognate peptide may also arise, in addition to T cells specific to the glyco-peptide, indicating that some antigens are deglycosylated during the priming of the immune response [62]. Thus, presumably, if an intact glycoprotein has been recognized via MGL, taken up by APCs, and transported to the endocytic pathway, the resulting (glyco-)peptides may appear in various forms, depending on the glycosylation pattern of the glycoprotein ingested, thereby affecting the subsequent preference of T cells for epitope recognition [67].

A different pattern of glycosylation can not only affect the antigen cleavage process and architecture of the presented antigen but also the signaling of second messengers. As MGL is able to oligomerize, it is also capable of binding multivalent forms of ligands, forming “endocytic synapses” [8,17,42,51]. A study performed by Napoletano et al. [57] showed that the interaction of MGL with the Tn-MUC1 glyco-peptide, with a high density of glycans (15 Tn residues), was stronger than MUC1 bearing lower amounts of GalNAc residues. Moreover, the density of GalNAc may be related to cross-talk through Ca^2+^—a crucial regulatory messenger for many biological processes. As in the case of other C-type lectins, the binding of a specific ligand by MGL is Ca^2+^ dependent. Calcium ions are required for interaction with the ligand and the stabilization of the carbohydrate recognition domain (CRD). The high engagement of the MGL receptor (high occupancy), due to the availability of a large amount of ligands (high concentration and frequency of sugar residues), induces Ca^2+^ influx at a high rate [38]. Intracellular calcium levels have been shown to be able to drive the DC phenotype from activation to tolerance [54]. It has been reported that an increase in intracellular Ca^2+^ is a signal for the production of IL-10, the main immunosuppressive interleukin, whereas a low concentration of intracellular Ca^2+^ resulted in the production of pro-inflammatory IL-12 [54,68]. It follows that the transport of calcium ions by an endocytosis of the multivalent MGL ligand may contribute to reduction in IL-12 production in favor of IL-10 synthesis, thus contributing to the switch to an immunosuppressive profile.

Although MGL is considered to be a receptor with immunosuppressive properties, there are some reports that prompt the speculation that the type of ligand recognized by it may diametrically influence the type of activated immune pathway. Although such assumptions need to be verified in vivo, it is fascinating that seemingly small changes in the configuration and the amount of single sugar residues located on the appropriate protein carrier can have such a powerful effect on the launching of a cascade of reactions, allowing for an appropriate immune profile to be attained. The different contexts of MGL ligands and their possible impacts on the immunological effect of MGL binding is summarized in Figure 2.

## 3. Molecular Basis of Ligand Recognition by MGL

### 3.1. Structural Matching of the Ligand

**Figure 3:**
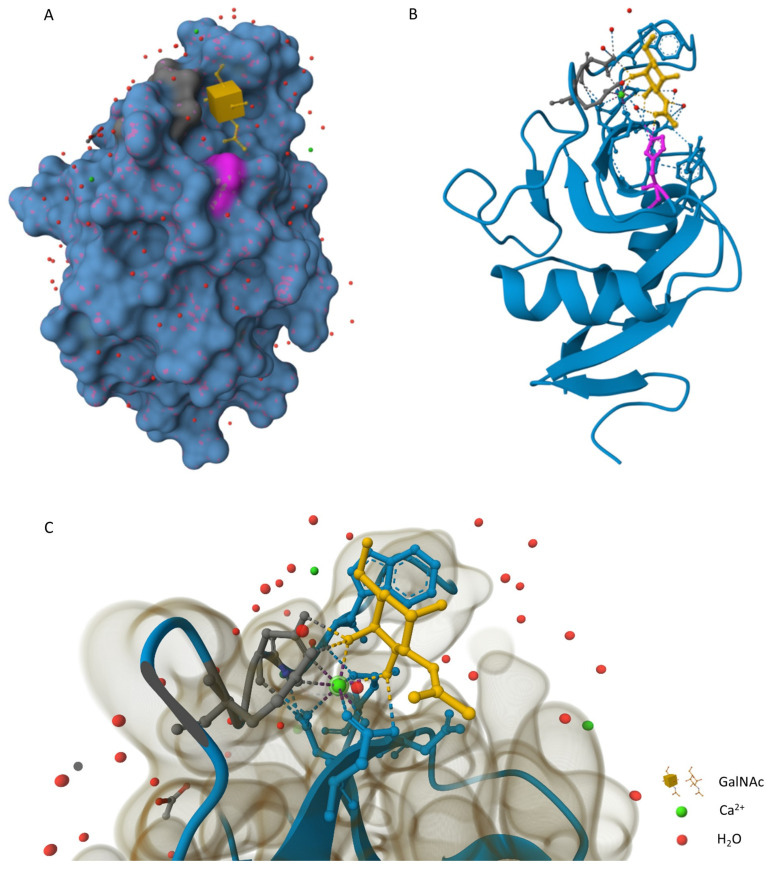
Crystal structure of the carbohydrate recognition domain of the human MGL bound to GalNAc: (**A**) surface representation and (**B**) cartoon representation, including interactions between GalNAc and CRD; (**C**) representation indicating GalNAc interactions with the primary binding site of MGL through the coordination of hydroxyl groups at C3 and C4 to the calcium ion. The QPD motif is colored in grey; the position of His^286^ is colored in pink. The images were created from the PDB structure 6PY1 [71] with the Mol*Viewer [76,77].

Like other CLRs, the structure of MGL contains a carbohydrate recognition domain, which facilitates the binding of the glycan moiety. Within the CRD, MGL displays the characteristic QPD (Gln-Pro-Asp) motif at the long loop region, which is responsible for sugar specificity (Figure 3A,B) [8,17,69,70,71,72]. MGL binds its ligand through the coordination of hydroxyl groups at C3 and C4 in GalNAc to the chelated calcium ion (Figure 3C) [73]. GalNAc substituted at these positions is not able to interact with MGL at the primary binding site [15]. However, the extension at C6 does not seem to interfere with MGL ligand recognition; thus, the 6-sialylated form of the Tn antigen is able to be bound [16,74]. MGL has multiple splice variants, which differ in the number of amino acids in a polypeptide structure. Higashi et al. [14] found seven isoforms in DCs, all of which include the endocytic YENF motif in the amino acid sequence of the cytosolic domain. The most abundant DC-expressed isoforms of MGL are short MGL 6C and long MGL 6B splice variants. To verify whether isoforms generated by alternative splicing also have distinct ligand binding properties, Marcelo et al. [70] attempted to compare the carbohydrate specificity of these main two MGL variants. Based on previous findings indicating Thr278 in a mouse MGL ortholog (MGL1) as crucial for sugar preference—Lewis X (Galβ1–4(Fucα1–3)GlcNAc), the authors additionally applied a histidine-to-threonine mutant of human MGL 6C in their studies. The mutant was constructed by changing His286 (His259 in the sequence of recombinant MGL 6C) to Thr in the CRD of human MGL, which corresponds to Thr278 of murine MGL1.

The two MGL subtypes (short MGL 6C and long MGL 6B) showed similar, but not identical, binding properties. Despite the presence of an identical amino acid sequence of CRD, some slight differences in the preferences of binding were observed [70]. The neck region of the short variant of MGL tends to oligomerize, forming homotrimers—clusters of binding sites preferring abundantly exposed glycans [75]. It has been suggested that the additional 27 amino acids in the stalk region of the longer MGL variant might potentially impede oligomerization. As this speculation has not been proven, it was assumed that the 27-amino-acid region may influence the orientation and spacing of MGL CRDs and, consequently, the range of recognizable ligands [70,75]. Unlike wild-type MGL, the histidine–threonine mutant was unable to interact with the sialylated Tn antigen. Even though the mutated variant could still bind a single GalNAc monosaccharide, its binding ability towards the Tn-containing glycopeptide had been lost. Moreover, the MGL mutant was unable to recognize Tn epitopes on tumor cell lines or its previously established Tn-containing ligands such as MUC1 or mucin 2 (MUC2) [70]. An interesting effect was observed, comparing the affinity of the mutated MGL toward GalNAcβ1-4Gal and αGalNAc. The binding capacity of the MGL mutant toward GalNAcβ1-4Gal was 10-fold lower compared to αGalNAc, which suggests that the first epitope may engage the secondary binding site for sufficient binding [51,52]. Moreover, based on a crystal model of a molecular arrangement of human MGL CRD and its sugar ligand, a secondary binding site containing His286 may also potentially fine-tune specificity to GalNAc residue. As histidine commonly forms H-bonds and water-mediated interactions, His286 may potentially participate in forming additional H-bonds with the 2-acetamido group (NHAc) of GalNAc (Figure 3B) [71].

Collectively, the results discussed in this section show that MGL not only binds to the carbohydrate epitope but may also engage the underlying peptide. Moreover, the data presented prompt the speculation that the MGL molecule has a secondary binding site within the CRD domain; when the primary site binds the carbohydrate GalNAc residue, the secondary site affects specificity to GalNAc and is necessary to connect to the peptide backbone or recognize elongated MGL ligands [52,70].

### 3.2. MGL-Mediated Metabolic Shifts

The molecular aspects of MGL-mediated interactions and signaling cascades that lead to DC stimulation are still not completely understood, but a recent study revealed some interesting findings. Zaal et al. [52] observed the impact of MGL activation on the metabolism of DCs. They investigated the effect of MGL engagement at the transcriptional level. Based on earlier evidence pointing to the different binding capacities of MGL for ligands, which differently exposed the GalNAc moiety, the authors applied two glycodendrimers (already mentioned in the previous sections), which contained an alpha-linked GalNAc residue (αGalNAc) or extended beta-linked GalNAc (GalNAcβ1-4Gal) [51,70]. As part of the research, DCs were stimulated with MGL ligands, followed by RNA sequencing analysis. Differential expression analysis showed that MGL stimulation by α-GalNAc had a minimal effect on gene expression compared to GalNAcβ1-4Gal. The authors also confirmed previous findings, showing that the MGL-mediated production of IL-10 requires concomitant TLR stimulation [37,43]. However, surprisingly, the effect of MGL engagement on gene expression was much smaller in the presence of lipopolysaccharide (LPS) as the TLR4 ligand than in the absence of TLR stimuli. Among the significant alterations in gene expression demonstrated by these studies, many terms/genes were confirmed to be involved in MAPK signaling, including ERK1/2, JNK, and p38 signaling. In addition, the analysis showed that the engagement of MGL alters the expression of genes involved in nuclear factor NF-κB signaling, G1/S phase transition, and neutrophil and leukocyte chemotaxis [52]. These findings appear to be consistent with the previous study performed by Napoletano et al. [45], demonstrating that MGL engagement activates extracellular signal-regulated kinase 1,2 (ERK1,2) and affects the NF-κB signaling pathway and T cell differentiation. Furthermore, an RNA sequencing analysis, after MGL binding to GalNAcβ1-4Gal, which was performed by Zaal et al. [52] revealed alterations concerning genes involved in glycolysis, oxidative phosphorylation (OXPHOS), and the tricarboxylic acid (TCA) cycle. This included enzymes such as hexokinase 3 (*HK3* gene), glucose-6-phosphate isomerase (*GPI* gene), glyceraldehyde-3-phosphate dehydrogenase (*GAPDH* gene), fructose-bisphosphate aldolase A (*ALDOA* gene), isocitrate dehydrogenases (*IDH2*, *IDH3B*, *IDH3G* genes), and NADH dehydrogenases (*NDUF* gene). To evaluate whether the expression changes in enzymes involved in glycolysis and oxidative phosphorylation actually affect energy metabolism, Zaal et al. [52] compared the metabolic activity of GalNAcβ1-4Gal- and αGalNAc-stimulated moDCs to unstimulated moDCs. A decrease in the glycolytic activity of stimulated moDCs in an MGL-dependent manner was recorded without significant differences in mitochondrial respiration. The effect was significant for both GalNAcβ1-4Gal- and αGalNAc-stimulated moDCs. These discoveries lead to very interesting conclusions, especially when considering the results of the research from the last decade, which demonstrated that changes in cellular metabolism has a great impact on DC development and function.

DC metabolism research revealed that, in inflammatory states, an increase in glycolysis is essential for the activation of immunogenic murine DC, which is associated with increasing biomass for effector function. In turn, reduction in glycolytic activity strongly limits the activation and lifespan of DCs after stimulation via TLRs [78,79]. Conversely, tolerogenic signals lead to a metabolic shift of human DCs towards OXPHOS and favor FAO (fatty acid oxidation), which may be related to energy recruitment for active suppression [80,81,82]. Also, the metabolism of pro-inflammatory macrophage subtypes is characterized by enhanced glycolysis, whereas macrophages with an anti-inflammatory phenotype mainly produce ATP through an oxidative TCA cycle coupled to oxidative phosphorylation and show lowered glycolysis [83]. Thus, the metabolic profile of immune cells, shifted towards OXPHOS with a decrease in glycolysis, has been associated with an anti-inflammatory and tolerogenic phenotype. It seems that the MGL-dependent metabolic reprogramming of DCs mediates this effect. However, the metabolic shift noted in MGL-stimulated DCs does not completely match this anti-inflammatory phenotype, as no substantial differences in oxidative mitochondrial activity were observed in DCs stimulated by MGL, in contrast to the tolerogenic immune cells previously analyzed [80,81,82]. It has been hypothesized that engagement of MGL on human DCs might silence a pro-inflammatory state within the DCs.

Taken together, it seems possible that the type of exposure of the terminal GalNAc residue may translate into binding efficiency and secondary binding site engagement and, as a consequence, may translate into the activation of a different type of molecular pathway, affecting the gene expression involved in the regulation of immune cell metabolism. Undoubtedly, there is a need for further research at the level of molecular interactions resulting from MGL ligand recognition.

## 4. Self and Non-Self Ligands of MGL

To date, it has been shown that, under normal physiological conditions, the presence of the endogenous Tn antigen is mainly restricted to CD45 on effector T cells and the embryonic brain [22,26]. Glycoepitopes potentially recognized by MGL may also be present on gangliosides GM2 and GD2 and on lymphatic endothelial structures in lymph nodes and thymus [15,84], but in general, the exposition of terminal GalNAc in healthy human tissues is not common, rather being attributed to pathological conditions, e.g., cancers in the form of Tn antigens (known as tumor-associated carbohydrate antigens—TACAs) [85]. Thus, it seems that MGL recognizes a limited array of self-ligands in healthy tissues. However, there may be some unidentified MGL ligands in the human body that remain to be discovered.

As overexpression of MGL occurs on tolerogenic APCs, and its interaction with the Tn antigen on the CD45 molecules of effector T cells results in a suppression of the inflammatory immune response; MGL is considered to play an important role in the maintenance of immune homeostasis [25]. The question arises as to whether, in the same line, cancer cells presenting terminal GalNAc residue in the form of Tn antigens or some pathogens known to be capable of exposing terminal GalNAc residues on bacterial LPS, viral envelope, or the shell of helminths can interact with MGL and influence the host’s immune response.

### 4.1. Cancer Cells

Tumor cells demonstrate a wide range of glycan alterations, and one of the main mechanisms underlying cancer-associated changes in glycosylation is the synthesis of incomplete structures such as the Tn/sTn antigen [86]. The Tn antigen, which consists of a single residue of GalNAc, is normally extended by glycosyltransferases to form longer and more diverse O-glycans [18,19,20,21,87]. The presence of Tn antigens has been demonstrated in approximately 90% of all cancers, and its overexpression promotes cancer cell proliferation and invasiveness and has been correlated with cancer progression and poor prognosis [26,88,89,90].

Early reports indicating the abundant occurrence of Tn antigens in cancer cells and its correlation with tumor invasiveness appeared in the 1980s and 1990s [18,20,21,91]. Over the last two decades, there have been several reports that Tn antigens present in various types of cancer are ligands recognized by MGL [15,16,17].

#### 4.1.1. Ligand Recognition by MGL and Immunosuppression in Cancers

There are some reports indicating the correlation of MGL ligand expression in tumors with some known oncogenic mutations, which are implicated in the development of immunosuppressive effects. It has also been revealed that identified MGL ligands in cancers co-occur with known immunosuppressive factors that have been previously associated with enhanced tumor growth. Although the exact network of interactions underlying these phenomena is not fully understood, this data may suggest that interactions involving MGL may constitute a part of the survival strategy of cancer cells.

Reports on MGL-mediated interactions with various types of cancer are presented below, as well as data indicating the possible effect of this interaction.

- Colorectal cancer

MUC1 is one of the best characterized tumor antigens, occurring in a broad spectrum of carcinomas. Normally, MUC1 is expressed on apical surfaces of glandular epithelial cells; during malignant transformation, its expression is highly upregulated and glycans are severely aberrant. A MUC1 containing Tn antigen expressed in colon cancer has been identified as an MGL ligand. The MGL binding is highly selective for tumor MUC1—MGL interacts with cancer-associated MUC1 but does not recognize MUC1 from normal cells [47,92].

A more recent study revealed a wider range of MGL-binding proteins from three different colorectal cancer cell lines. Among the major MGL ligands, different classes of proteins have been found, including cell surface signaling receptors and integrins but also proteins whose function is not precisely defined.

Glycopeptide analyses detected both LacdiNAc and Tn epitopes on proteins that bound MGL in colorectal cancer, representing known target ligands of MGL [93]. However, what is interesting is that N-linked glycans (LacdiNAc) played a major role in this interaction [94].

It has been demonstrated that a high expression of MGL ligands is associated with the lower disease-free survival of patients with stage III colorectal cancer. Moreover, MGL ligand expression has a positive correlation with BRAF mutation. BRAF is a component in the MAPK (ERK) signaling pathway, involved in the activation of transcription factors important for cell growth, proliferation, and survival [95,96,97,98]. Both MGL engagement and oncogenic BRAF mutations have been also implicated in the development of the immunosuppressive effect. It has been proposed that oncogenic alterations involved in MAPK signaling, including BRAFV600E mutations, may induce MGL ligand expression and alteration in the tumor cell glycosylation profile [47].

- Breast cancer

HER2/neu is another molecule controlling cell growth, differentiation, and migration, whose expression is associated with high MGL-binding molecule levels in breast cancer cells [99]. HER2 is involved in the activation of phosphatidylinositol triphosphate kinase (PI3K) and MAPK signaling [100]. These associations are consistent with the hypothesis that various oncogenic alterations involved in the activation of the MAPK pathway may lead to aberrations in tumor cell glycosylation and the enhanced expression of MGL ligands, influencing immunosuppression [47].

However, more recently, Kurze et al. [101] observed that the increased expression of the Tn antigen in breast cancer tissues led to an increased MGL-mediated phagocytosis by macrophages, which in turn improved disease-free survival due to the uptake of damaged and dead cells.

- Cervical cancer

MGL ligand expression is correlated with lower survival and distant metastasis in cervical cancers. Furthermore, MGL ligand expression is associated with PIK3CA mutations, the most frequent oncogenic mutations in cervical cancer [102]. The PIK3CA gene encodes the catalytic subunits of PI3K, which are able to activate the PI3K/AKT/mTOR pathway [103,104,105]. The PI3K/AKT/mTOR pathway is involved in tumor growth control including proliferation, metastasis, angiogenesis, and apoptosis evasion [106]. In many cancers, this pathway is overactive and, interestingly, it has been shown to be implicated in glycosylation pathways [107]. Thus, the PI3K/AKT/mTOR pathway could possibly be involved in enhancing MGL ligand expression, especially since mutations in MAPK signaling genes, which—as already mentioned—have also been correlated with the expression of MGL ligands, constitutively activate the PI3K pathway [102,106]. However, further investigation is needed to confirm this causative signaling network.

Moreover, in cervical cancer patients, MGL low ligand expression was associated with a high amount of CD14+ myeloid cells, which in turn has been correlated with a high frequency of cytotoxic T cells and prolonged survival. Conversely, an enhancement of MGL ligands expression has been associated with the presence of CD14-CD163+ myeloid cells, which in turn has previously been correlated to high number of regulatory T cells and poor prognosis [108]. Thus, the inverse relationship between MGL and CD14+ myeloid cells may be related to a cancer-specific survival strategy [102].

- Glioblastoma

A high occurrence of both the MGL receptor on immunosuppressive CD163+ tumor-associated macrophages and GalNAc-terminated glycans as its ligands has also been demonstrated in glioblastoma patient-derived tumor tissues. An in vivo murine model showed that the overexpression of MGL ligands in glioblastoma leads to an increased frequency of PD-L1+ macrophages in tumors, which has been speculated to play a major role in suppressing the adaptive arm of immunity [109].

- Ovarian cancer

Expression of Tn/sTn has also been corelated with the increased tumor aggressiveness of ovarian cancers. Napoletano et al. [110] attempted to study O-glycoproteome in ovarian cancer and identify the potential carriers of Tn/sTn antigens, which were possibly relevant in tumor–DC interactions through the MGL receptor. Based on the experimental model applied, the authors speculated that there was a selective MGL specificity to the Tn antigen. Interestingly, the authors found that MGL and VVA (lectin from *Vicia villosa*)— lectins having the same glycan moiety specificity—had distinct reactivity profiles. In addition to the exposure of the terminal GalNAc residue, the binding of MGL in ovarian cancer cells is influenced by other factors, such as glycopeptide conformation and glycan arrangement along the peptide backbone. This high restriction of MGL binding may be due to the physiological role of MGL, which limits the repertoire of glycoproteins efficiently interacting with MGL to trigger an effective immune effect. Some of the glycoproteins identified as MGL binders have already been linked to ovarian cancer progression which may suggest the participation of MGL–ligand interaction in providing a pro-carcinogenic immune microenvironment [110].

- T cell leukemia

The T cell leukemia model cell line Jurkat is known to have a high level of Tn antigens due to a mutation in Cosmc. Acute T-cell leukemia cells have been utilized as a model system to study the immunoregulatory properties of MGL and the effect of its ligand recognition [24,111]. CD43 and CD45 have been described as the main carriers of the Tn antigen interacting with MGL in Jurkat cells [24]. This interaction results in an anti-inflammatory response, thus reducing the proliferation of T-cells and induction of cell death in Jurkat. More recent research revealed a wider set of potential MGL-binding proteins of Jurkat cells. Apart from CD43 and CD45, the authors identified 15 proteins potentially interacting with MGL, including transmembrane signaling molecules such as semaphorin-4D or tumor necrosis factor receptor superfamily member 8 (TNFRSF8) [112]. Similar to the study by Napoletano et al. [110] on the O-glycoproteome of ovarian cancer, the results obtained by Pirro et al. showed high MGL selectivity for its ligand—the presence of the Tn antigen was not sufficient for binding [112]. Many functions of the newly identified potential MGL ligands are unknown, especially in the context of immune response. This points to a new area of research, because, although there is evidence on the immunoregulatory role of MGL in tumor cells, further investigations should evaluate the functional effect of interactions mediated by selected proteins in cancer–immune responses [112].

#### 4.1.2. MGL-Mediated Interactions as a Potential Anti-Cancer Strategy

Although many cancer-related studies on MGL interactions may confirm their immunosuppressive properties, there are several recent reports indicating a correlation between low MGL expression and worse outcomes of cancer patients, including hepatocellular carcinoma, melanoma, lung adenocarcinoma, breast cancer, and head and neck squamous cell carcinoma [113,114,115,116,117,118]. In addition to the association between the low expression of MGL and poor prognosis in cancer patients, the study showed that the MGL level is significantly correlated with immune infiltration in the tumor microenvironment, which may be a direct explanation for its possible protective effect in some types of tumors [115,116,117,118]. The studies mentioned above are mainly based on bioinformatics analysis on data acquired from online databases and artificial intelligence algorithms and thus require experimental and clinical verification. Nevertheless, the data obtained may somehow suggest the dualistic role of MGL acting as a double-edged sword. As an endocytic receptor expressed on antigen-presenting cells, MGL may potentially participate in an anti-tumor defense via antigen uptake and presentation, T cell activation, and the initiation of a specific immune response [69]. On the other hand, tumor cells under certain conditions may outwit the host strategy and create an immunosuppressive environment by exploiting the immune-regulatory properties of MGL, thus allowing for efficient cancer cell proliferation and metastasis.

To summarize cancer-related MGL binding, an interaction with MGL has been described for many types of cancers including colon cancer, breast cancer, cervical cancer, glioblastoma, ovarian cancer, or acute T-cell leukemia. For some types of cancer, the expression of MGL ligands correlates with frequent oncogenic mutations associated with tumor cell proliferation, metastasis, and immunosuppressive effects, as well as with the lower disease-free survival of patients. These data suggest that MGL ligands may be directly related to oncogenic transformation, and MGL-mediated interactions are part of the cancer survival strategy. However, MGL binding shows high restriction as in many cases—the Tn antigen is not sufficient for an interaction with MGL. The interaction may be influenced by other factors. There are also some recent bioinformatics analyses suggesting that MGL may serve a protective role in some types of tumors, indicating its dualistic role in influencing immune response. However, it seems that there is still much to be discovered in this field.

### 4.2. Pathogens

CLRs have evolved to differentiate ligands as self or non-self structures. However, these interactions do not always remain supportive to the host immune strategy. Some bacteria exploit these receptors to evade host immune response. The expression of hMGL on APCs residing in different locations of the human body [84] enables the detection of pathogens exposing the GalNAc epitope (e.g., by bacterial LPS, viral envelope, or the shell of helminths) within the tissues. As different tissues are populated with different subtypes of APCs containing distinct specific immunological markers, the functional effect of MGL engagement, in addition to the type of ligand, may also depend on the tissue or location where the interaction occurs [6]. As some relevant MGL-mediated interactions in infectious diseases have been tested on mice models, this section summarizes microbial ligands for human MGL (hMGLs) as well as mice MGLs (mMGLs) and the immunological outcome of their recognition.

#### 4.2.1. Bacteria

- *Neisseria gonorrhoeae*

*Neisseria gonorrhoeae* is an agent of gonorrhea, a sexually transmitted infection. *N. gonorrhoeae* primarily colonizes and invades genital mucosa [119]. *N. gonorrhoeae* lipooligosaccharide (LOS) phenotype C, carrying a terminal GalNAc, is the first bacterial ligand to be described for hMGL. Interestingly, there are different stable gonococcal LOS phenotypes that interact with human dendritic cells, with each variant targeting a different set of receptors [120]. The binding of hMGL on human moDCs through bacteria shifts DC cytokine secretion to direct T helper cell differentiation toward Th2 and Th17 profiles [6,120,121]. The Th17 phenotype is less capable of inducing a protective response against *N. gonorrhoeae* than Th1 [122,123]. Considering the presence of DCs carrying MGL at the female and male genital tract, it seems that hMGL-mediated phenotypic changes in DCs that lead to immune polarization toward a less effective Th profile may be a part of the gonococcal strategy to escape immunosurveillance during an infection of the genital tract.

- *Campylobacter jejuni*

*Campylobacter jejuni* is mainly a gastrointestinal pathogen and cause of foodborne infections [124]. Important virulent factors of *C. jejuni* include the polysaccharide capsule and the presence of a unique protein glycosylation (pgl) gene cluster, which regulates the N-linked glycosylation of bacterial glycoproteins [125]. *C. jejuni* N-linked glycan has been described as containing one D-N′,N′-diacetylbacillosamine (Bac) and five D-N-acetylgalactosamines with a D-glucose (Glc) branch (Figure 1) [126,127]. Pgl-dependent glycosylation has also been described as mediating interaction with hMGL. The loss of specific structures containing terminal GalNAc residue abrogates hMGL binding and induces pro-inflammatory cytokine production by moDCs [6,128].

- *Staphylococcus aureus*

*Staphylococcus aureus* is a skin resident but often also the cause of skin infections [129]. *S. aureus* ST395 lineage produces unique wall teichoic acid (WTA) with α-O-N-acetylgalactosamine residues (GalNAc) and is another microbe that can be detected by hMGL [130,131,132]. Surprisingly, interaction between the *S. aureus* ST395 strain and hMGL expressed on DCs and macrophages induces DC maturation and enhances the production of cytokines, especially IL-6 and IL-12p70. This suggests that the binding of S. aureus WTA glyco-epitope by MGL is part of the immune defense strategy and not part of the immune evasion of *S. aureus* [132,133].

- *Bordetella pertussis*

The lungs are sites of infection for many pathogens, including *Bordetella pertussis.* As the respiratory tract is highly exposed to many microbes, the human organism has developed several strategies of immune defense in this location. The respiratory tract is populated with many subsets of APCs. In the lungs, the essential line of immune defense is mainly formed by macrophages and dendritic cells. However, mast cells have also been reported to be involved in fighting invading pathogens. It has been demonstrated that mast cells are able to interact with *B. pertussis,* present its antigens to T cells, and induce cytokine secretion [134]. It has been suggested that such interactions may be mediated by MGL, which is able to bind the LOS of *B. pertussis*. This interaction was analyzed on mouse mast cells, which were stimulated with heat-inactivated *B. pertussis* or the isolated LOS of *B. pertussis*. Analyses showed that mast cells stimulated in this way induce the production of TNF-α, IL-6, and INF-γ, which was inhibited by blocking MGL. This result suggests the possible role of MGL in the binding and uptake of *B. pertussis* antigens [23,135,136]. However, this interaction was analyzed on a mouse mast cell model. As already mentioned, there is only one ortholog of human MGL, whereas in mice, two distinct MGL orthologs are present—mMGL1 and mMGL2—showing different affinities [23]. In the experiment, a dual-specific blocking antibody was used, and, as a consequence, binding could not be attributed to either mMGL1 or mMGL2. Thus, this study cannot determine which lectin participates in *B. pertussis* recognition (by mast cells) and whether it is reflected in the human organism.

- *Mycobacterium tuberculosis*

*Mycobacterium tuberculosis* is the cause of tuberculosis—a primary lung infection [137]. Recently, Naqvi et al. [138] documented the important antibacterial and regulatory role of mMGL1 immunity to *M. tuberculosis*. Their research demonstrated mMGL activation in response to *M. tuberculosis* exposure, its upregulation, and the accumulation of mMGL-expressing cells at sites of mycobacterial-driven inflammation in the lungs. Importantly, a deficiency of mMGL1 promotes the production of pro-inflammatory cytokines (IL-1β, IL-6, IFN-γ), exacerbates inflammation, and increases lung mycobacterial burden. Moreover, the study by Naqvi et al. [138] showed an increased accumulation of host lipids in the pulmonary macrophages of mMGL1-deficient mice infected with *M. tuberculosis* compared with wild-type (WT) mice. An accumulation of host lipids has also been described as an important factor in the pathogenesis of tuberculosis, as they serve as nutrient sources for the pathogen and modulators of the host immune response [138,139,140]. The pathogen-specific moieties that mMGL binds in *M. tuberculosis* have not yet been defined, and there are no published data to confirm similar mechanisms in humans. However, these results indicate that the ability of MGL to regulate or silence the pro-inflammatory response may play a role in antimicrobial immunity. Moreover, studies indicate that the action of MGL may involve the regulation of lipid metabolism.

- *Klebsiella pneumoniae*

A similar mechanism has been previously described for another lung infection, caused by *Klebsiella pneumoniae*. As in the case of *M. tuberculosis* infection, mMGL1 was upregulated in immune cells that infiltrated the lungs of a murine model of pneumonic sepsis caused by *K. pneumoniae*. The silencing of mMGL1 resulted in significantly increased mortality from infection compared to mMGL1-sufficient wild-type mice. Moreover, mice with mMGL1 deficiency showed a hyperinflammatory response, massive pulmonary neutrophilia, and an increase in neutrophil-associated murine immune mediators. All of these findings suggest the important role of MGL in the resolution of inflammation in a lung microenvironment. However, this result concerns interaction with mMGL1-binding Lewis X epitopes rather than terminal GalNAc and did not account for the hMGL ortholog [141].

#### 4.2.2. Helminths

- *Schistosoma mansoni*

The current state of knowledge strongly points to the crucial contribution of sugar antigens to the immunobiology of schistosomiasis caused by *Schistosoma mansoni*. *S. mansoni*, entrapped in the liver of the host, secretes soluble egg antigens (SEA), which contain a wide range of glycopeptides that are able to bind CLRs, including MGL [142,143,144]. SEA suppresses Th1 responses, skewing immunity toward the Th2 profile [145]. Such Th2 deviation often leads to parasite host immune evasion but also serves as a protective mechanism for the host to survive the infection [146]. It has been demonstrated that the engagement of hMGL expressed by iDCs through terminal GalNAc residues on the *S. mansoni* egg contributes to the induction of the Th2 response [15,147]. Human monocyte-derived immature DCs stimulated with SEA do not undergo a conventional maturation process in vitro. However, CLRs, including hMGL, are able to participate in the internalization of SEA and co-localization with MHCII in lysosomal compartments. These results suggest that SEA internalization through CLRs on DCs leads to processing and antigen presentation to T cells and may affect Th1/Th2 balance in favor of Th2 [147].

- *Trichuris suis*

*Trichuris suis* is another parasite that, by binding its soluble product (SP) glycans with CLRs, influences phenotypic changes in human DCs. *T. suis* is not a human parasite, but its SPs are currently used in therapeutic approaches to inflammatory diseases because of their ability to suppress LPS and the TNF-α induced secretion of pro-inflammatory mediators. The SPs of *Trichuris suis* affect the production of cytokines, suppressing those with pro-inflammatory properties, including IL-12, TNF-α, and IL-6, as well as pro-inflammatory chemokines [148]. Such an effect, accompanied by an overexpression of OX40L and CXCL16, is a trigger for Th2 polarization [148,149,150]. Such an effect has been proposed as being attributed to interactions of SPs with CLRs, including MGL. The ability to bind GalNAc-terminated glycans of SPs through MGL has been assessed using recombinant hMGL. However, an in vitro study revealed a lower contribution of hMGL in SP binding to moDCs compared to other analyzed CLRs.

- *Fasciola hepatica*

The liver fluke *Fasciola hepatica* causes fasciolosis. In the case of human fasciolosis, a parasitic organism perforates the liver and migrates toward biliary radicles [151]. *F. hepatica’s* strategy to survive in the host organism and evade host immune control also largely depends on glycan-mediated Th2/Treg deviation [152,153,154,155,156,157]. *F. hepatica* expresses an O-linked Tn antigen [158], with a proven ability to interact with hMGL and, as a consequence, to modulate the TLR-2-induced maturation of human monocyte-derived DCs (mo-DCs) through the enhanced production of IL-10 and TNF-α. Moreover, it has also been demonstrated that mMGL2-expressing cells are recruited to the peritoneum in *F. hepatica*-infected mice and produce many regulatory cytokines such as IL-10, TNF-α, TGF β, and other factors promoting Th2 immune responses while suppressing Th1 polarization [50]. In addition, the production of immunomodulatory molecules by mMGL2+ cells is associated with an increase in clinical symptoms and Treg expansion. Thus, it seems that MGL interactions are *F. hepatica’s* strategy to escape host immune control [159].

- *Taenia crassiceps*

*Taenia crassiceps* is a cestode parasite that rarely infect humans, but there are reports demonstrating that immunocompromised humans can develop cysticercosis caused by *T. crassiceps*. In infected humans, parasites accumulate in the subcutis and among muscular tissues [160]. In a model of experimental cysticercosis, the engagement of mMGL1 expressed by macrophages by *T. crassiceps* glycoconjugates demonstrated enhanced parasite clearance and positively regulated inflammation. The results presented by Montero-Barrera et al. [161] showed reduced intracellular macrophage signaling in response to *Taenia* antigens as well as lower TNF-α and nitric oxide (NO) production in mMGL1-deficient mice. These findings suggest the important role of mMGL1 in driving macrophage responses in vivo and mediating resistance to this helminth infection.

#### 4.2.3. Viruses

Many CLRs are exploited by viruses as a cellular entrance, which allows for viral replication. MGL has been suggested as serving as an entry receptor for the Ebola virus, SARS-CoV-2, or influenza virus [162,163,164,165,166]. However, no results so far support MGL signaling as contributing to any influence on immunological responses against virus infections.

The data discussed in the Pathogens section suggest that interactions with the known pathogen ligands of MGL may lead to different immunological effects. Some pathogens seem to evolve to modulate the host immune system through MGL interaction and promote their own survival. On the other hand, MGL binding serves as a host immune strategy for pathogen clearance in some cases. The interactions discussed are summarized in Table 1.

It seems that various factors can influence the type of immune response mediated by MGL. The final outcome of the immune response should be considered to be potentially influenced by the type of APC expressing MGL, as they contain distinct specific immunological markers within different tissues [6]. Thus, this prompts speculation that the site and microenvironment in which the interaction occurs may also affect the type of immune response. Undoubtedly, not all interactions have been tested in a model that would enable the assessment of whether a specific immunological effect is reflected in the microenvironment at the site of infection Although, it appears that, within the respiratory tract, APCs expressing MGL may have a significant role as concerns protection during the invasion of microorganisms, based on the data presented. It seems plausible when considering the respiratory tract’s high exposure to various airborne pathogens. The human body had to develop many lines of defense in this site to protect the organism against pathogenic microorganisms. The protective function of MGL during infection may also be based on the regulation of developing inflammation in response to infection, such as it has been reported for the MGL-mediated response to *K. pneumoniae* or *M. tuberculosis* [138,141]. This effect is consistent with the assumption that the primary role of MGL is to protect the organism from excessive inflammation and tissue damage. In the case of some pathogens, this function could be exploited as their own survival strategy. However, the data for respiratory infections concern the mMGL1 ortholog, which differs in sugar specificity from hMGL and may not be reflected in humans.

In case of infections caused by parasites, the contribution of microenvironment factors to the MGL-mediated immune response may be difficult to assess, as, depending on the developmental stage, they can migrate within different tissues but also exist in distinct host organisms. Nevertheless, it seems that MGL-mediated interactions favor parasitic survival.

## 5. MGL Interactions—Relevant for Fertilization and Pregnancy?

CLRs are able to interact with foreign ligands within the human organism and mediate the development of specific immune responses. However, not all interactions mediated by these receptors support host defense. Some pathogens and metastatic cancer cells have learned to use sugar–lectin binding to trick the immune system and survive. Likewise, specific glycan-mediated interactions seem to be important at early stages of human development.

The embryo and male gametes contain antigens that can trigger destructive immune responses in the female reproductive system, which must be overcome in order to ensure successful fertilization, implantation, and pregnancy. The mechanisms enabling the avoidance of maternal immunity and the development of maternal tolerance to the fetus are still not fully understood [167]. In the 1990s, Clark et al. proposed the hypothesis referred to as the human fetoembryonic defense system hypothesis (hu-FEDS), which suggests that the human fetus is protected during development by glycoconjugates presenting sugar residues, which are recognized by the mother’s immune system in order to evoke tolerance [27,29,30]. The model assumes that glycan-mediated interactions in the female reproductive tract during fertilization and pregnancy are similar to the mechanisms that pathogens and metastatic cancer cells use to evade host immune surveillance [28,31].

After coitus, the cervix is massively infiltrated by female immune cells, including DCs. Dendritic cells, as APCs, participate in the development of reactions directed against foreign antigens; on the other hand, they have the ability to activate immunosuppressive pathways, which makes them a key switch in the regulation of homeostasis. The subpopulation of DCs with a tolerogenic phenotype is pivotal in the activation and expansion of Treg cells, which are necessary during implantation [30,167]. Tolerogenic DCs display an immature or semi-mature phenotype, and their differentiation is mediated by immunosuppressive factors [168,169]. Seminal plasma has been shown to contribute to this process [30,167]. One of the assumptions of the hu-FEDS hypothesis is the involvement of glycans exposed by seminal plasma glycoproteins in immunomodulatory interactions in the post-coital period. Seminal plasma has been revealed to be abundant in glycans that are not commonly found in healthy tissues but are associated with carcinogenesis and bacterial pathogenesis. These glycans are potential ligands for the endogenous lectins of the immune system. Examples include high-mannose glycans and tri- and tetrasaccharides terminated with Lewis X and/or Lewis Y, which are known ligands for DC-SIGN. Lewis X/Y antigens are rarely found on the oligosaccharides present on the surface of other normal cells, but their high expression is also associated with cancers and is known to induce immunosuppression [27,31]. Moreover, DC-SIGN-mediated interactions are also utilized as an immune evasion strategy by some pathogens that express fucosylated Lewis type antigens, e.g., *Helicobacter pylori* or *Schistosoma* [170,171].

Our recent study revealed the glycoproteins of seminal plasma, which are potential carriers of O-glycans terminated with Gal and GalNAc residues, including T and Tn antigens [172]. As already mentioned, both T and its precursor Tn antigen are hidden in normal cells but selectively exposed on cancer cells [87]. The presence of these glycoepitopes on identified glycoproteins makes them candidates for ligands of endogenous galactose-specific lectins, such as galectins or MGL. As potential carriers of GalNAc-terminated glycans, which are preferred by the MGL receptor, our analysis indicated prolactin-inducible protein (PIP), semenogelin-1 (SEMG1), semenogelin-2 SEMG2, lactotransferrin (LTF), prostate-specific antigen (KLK3), and fibronectin (FN1) [172].

Although many interactions that are mediated by lectin receptors have been described as potentially relevant for fertilization and pregnancy, galectins have gained considerable attention. They have been shown to be involved in embryo implantation, placentation, angiogenesis, and the development of maternal immune tolerance [173]. However, there is no report on the involvement of MGL—another galactose-specific lectin—in reproductive-related interactions.

The mechanism of action of galectins appears to be complex. Galectins have the capacity to form lattices between glycoproteins on the surface of cells and between cells; therefore, they are able to promote a plethora of biological activities in many different biological pathways [174]. Although current evidence indicates that galectins have many functions in a healthy pregnancy, a full understanding of their mechanisms requires more research [173]. One of the interesting reports indicates that the galectin-1 present in decidua is one of the key regulators of tolerogenic DCs capable of inducing Treg cells [175]. Moreover, it has been revealed that galectin-1, secreted by uterine natural killer cells, induces the apoptosis of activated decidual T cells [176]. The ability to mediate primary-activated human T cells death has been also attributed to galectin-3, which is present in seminal plasma, indicating that it could induce immune deviations in female reproductive systems in the post-coital period [177,178]. T cell apoptosis, mediated by galectin-3 or galectin-1, can differ in many ways, but both galectins, similar to MGL, have the ability to bind effector cell surface glycoproteins, including CD45, and to influence the threshold of TCR signaling [179]. There are some similarities in ligand preferences, as well as the signaling pathway and immunological effects induced by MGL and galectins (e.g., as discussed for galectin-1 and galectin-3). Although there are no results so far confirming that MGL and galectins may crosstalk, it is worth considering, based on the similarities mentioned, whether these lectins could act in a signaling network to contribute a specific immune outcome.

Tn and sialyl Tn epitopes are known as oncofetal antigens [22]. The Tn antigen is known to be highly expressed by the embryonic brain, and the sialyl Tn antigen is expressed in fetal organs and the amniotic fluid [180,181,182,183]. Both glycoepitopes are included in the repertoire of MGL ligands, but there is no evidence so far for the participation of MGL in immune regulation in fertilization or pregnancy. However, in light of the assumptions of the hu-FEDS hypothesis and the interactions described for MGL suggesting that cancer cells and some pathogens may exploit the immunoregulatory function of MGL as part of a survival strategy, the presence of MGL-recognized glycans on fetal tissues, amniotic fluid, placenta, and seminal plasma strongly suggests the involvement of this lectin in immunosuppression and the development of maternal immunological tolerance towards foreign antigens.

Considering the data presented, MGL could potentially support immunosuppression and maternal tolerance at various levels:

- The engagement of MGL potentially expressed by iDCs in the cervix during the post-coital period might support the induction of the tolerogenic phenotype of DCs and modulate DC function;
- At different stages of fertilization and pregnancy, MGL might cross-talk with galectins to affect molecular pathways leading to effector T cell apoptosis and immunosuppression;
- During embryo development, Tn and sTn, highly expressed by fetal organs and amniotic fluid components, may target MGL to maintain immune homeostasis.

However, such assumptions are hypothetical and need to be verified. Therefore, the involvement of MGL in conception seems to be another area to explore, which is relevant in the context of infertility or recurrent miscarriages. Determining whether MGL and its ligands are involved in the modulation of the maternal immune response during fertilization or pregnancy and whether they participate in the development of maternal tolerance to the fetus may help in finding the cause of recurrent miscarriages or pre-eclampsia. It is possible that specific patterns of the expression of immunoregulatory factors in the amniotic fluid or semen, the means by which glycan moieties are exposed, and their ability to interact with lectins in the maternal immune system may affect the fertilization process or correlate with an abnormal course of pregnancy and be a potential predictor of miscarriages. 

## 6. Conclusions

Glycans present on cells and macromolecules of living organisms may act as biological information carriers. Simple and complex carbohydrates are recognized by many types of proteins, including lectins in the immune system. So far, several types of human immune system lectins have been described, such as I-type lectins (Siglecs), galectins, or C-type lectins (CLR). Specific to terminal GalNAc, MGL is a C-type lectin (CLR) receptor and is exclusively expressed on DCs and macrophages. The high level of MGL expression on APCs with a tolerogenic phenotype indicates its immunosuppressive properties. However, the structure of MGL ligands and context of their exposition may be important in promoting a specific immune response. The means of the exposure of the GalNAc residue and length of the glycopeptide carrying the carbohydrate ligand may influence the activation of a particular signal transduction pathway, the intracellular fate of the internalized ligand, and the metabolic profile of immune cells expressing MGL. Human MGL recognizes a limited array of self-ligands in healthy human tissues. Up to now, the presence of endogenous MGL ligands has been demonstrated as mainly restricted to CD45 on effector T cells and the embryonic brain under normal physiological conditions. The interaction of MGL expressed on tolerogenic APCs with the CD45 of effector T cells leads to the suppression of the inflammatory immune response. Therefore, MGL is considered to be a factor playing an important role in maintaining the homeostasis of the immune system and contributing to immune tolerance. MGL as a C-type lectin receptor is also able to uptake non-self antigens for the processing, presentation, and development of a specific immune response. The expression of MGL on APCs residing in different sites of the human body enables the detection of foreign ligands exposing the GalNAc epitope within the tissues. Some pathogens exploit the immunoregulatory properties of MGL to suppress inflammation or redirect a response toward less effective Th2 profiles, thus evading a host immune response. Tumors under certain conditions are able to induce an immunosuppressive environment through MGL engagement, enabling effective cancer cell proliferation and metastasis. Many data suggest that the expression of MGL ligands may be directly related to oncogenic transformation and be a part of the cancer survival strategy. However, it seems that various factors can influence the type of immune response mediated by MGL. The final effect of MGL engagement, in addition to the type of ligand, may also depend on the microenvironment and the site in the human body where the binding occurs. Research on these interactions may be helpful in understanding the molecular mechanisms of the pathogenesis of various infectious diseases or tumor metastasis, as well as in developing immunotherapeutic intervention. This article discusses several discoveries related to the recognition of various ligands by MGL; however, the complexity of these interactions at the molecular level and their diverse immunological outcomes make their final effect ambiguous, and further research in this field is needed. It seems that another interesting direction of study may be to define whether MGL-mediated interaction occurs during fertilization and pregnancy. The answer to the question as to whether MGL and its ligands are involved in the modulation of the response to male gametes, the embryo, and the fetus may help to expand knowledge about the immunological aspects of fertilization and pregnancy and may also be helpful in the search for the causes of unexplained infertility, recurrent miscarriages, or preeclampsia, which are currently becoming a more common serious medical and social problem.

## Figures and Tables

**Table 1:** Summary of MGL microbial ligands and immunological outcomes of their MGL-mediated recognition.

Species	Site of Infection/ Occurrence	Outcome of MGL-Mediated Interaction	Survival Strategy	
			Host (Immune Defense)	Pathogen (Immune Evasion)
**Bacteria**				
*Neisseria gonorrhoeae*	genital tract	Th2/Th17 polarization [120]		+
*Campylobacer jejuni*	gastrointestinal tract	Suppression of pro-inflammatory cytokine (IL-6) production [128]		+
*Staphylococcus aureus*	skin	DC maturation and enhanced production of pro-inflammatory cytokines [132]	+	
*Bordetella pertussis* *	respiratory tract (lung)	Production of TNF-α, IL-6, and INF-γ [135]	+	
*Mycobacterium tuberculosis* *	respiratory tract (lung)	Regulation of inflammatory response [138]	+	
*Klebsiella pneumoniae* *	respiratory tract (lung)	Regulation of inflammatory response [141]	+	
**Helminth**				
*Schistosoma mansoni*	liver	Th2 polarization [147]		+
*Trichuris suis*	not in humans	Th2 polarization [148]		+
*Fasciola hepatica*	liver	Th2/Treg deviation [50]		+
*Taenia crassiceps* *	subcutis; muscular tissues	Promotion of inflammatory response, enhanced TNF-α and NO production [161]	+	

* Interaction tested with the mouse ortholog, not confirmed with human MGL.

## Data Availability

Not applicable.
